# Feasibility of precision smoking treatment in a low-income community setting: results of a pilot randomized controlled trial in The Southern Community Cohort Study

**DOI:** 10.1186/s13722-024-00441-1

**Published:** 2024-03-15

**Authors:** Scott S. Lee, Nicole Senft Everson, Maureen Sanderson, Rebecca Selove, William J. Blot, Stephen King, Karen Gilliam, Suman Kundu, Mark Steinwandel, Sarah J. Sternlieb, Qiuyin Cai, Shaneda Warren Andersen, Debra L. Friedman, Erin Connors Kelly, Mary Kay Fadden, Matthew S. Freiberg, Quinn S. Wells, Juan Canedo, Rachel F. Tyndale, Robert P. Young, Raewyn J. Hopkins, Hilary A. Tindle

**Affiliations:** 1 Vanderbilt University Medical Center, 2525 West End Ave. Suite 450, Nashville, TN 37203 USA; 2 Meharry Medical College, Nashville, TN USA; 3 Tennessee State University, Nashville, TN USA; 4 University of Wisconsin-Madison, University of Wisconsin Carbone Cancer Center, Madison, WI USA; 5 Geriatric Research Education and Clinical Centers (GRECC), Veterans Affairs Tennessee Valley Healthcare System, Nashville, TN USA; 6 Centre for Addiction and Mental Health, and Departments of Pharmacology & Toxicology, and Psychiatry, Campbell Family Mental Health Research Institute, University of Toronto, Toronto, ON Canada; 7 University of Auckland, Auckland, New Zealand

**Keywords:** Smoking cessation treatment, Precision medicine, Health disparities

## Abstract

**Background:**

The feasibility of precision smoking treatment in socioeconomically disadvantaged communities has not been studied.

**Methods:**

Participants in the Southern Community Cohort Study who smoked daily were invited to join a pilot randomized controlled trial of three smoking cessation interventions: guideline-based care (GBC), GBC plus nicotine metabolism-informed care (MIC), and GBC plus counseling guided by a polygenic risk score (PRS) for lung cancer. Feasibility was assessed by rates of study enrollment, engagement, and retention, targeting > 70% for each. Using logistic regression, we also assessed whether feasibility varied by age, sex, race, income, education, and attitudes toward precision smoking treatment.

**Results:**

Of 92 eligible individuals (79.3% Black; 68.2% with household income < $15,000), 67 (72.8%; 95% CI 63.0–80.9%) enrolled and were randomized. Of these, 58 (86.6%; 95% CI 76.4–92.8%) engaged with the intervention, and of these engaged participants, 43 (74.1%; 95% CI 61.6–83.7%) were retained at 6-month follow-up. Conditional on enrollment, older age was associated with lower engagement (OR 0.83, 95% CI 0.73–0.95, p = 0.008). Conditional on engagement, retention was significantly lower in the PRS arm than in the GBC arm (OR 0.18, 95% CI 0.03–1.00, p = 0.050). No other selection effects were observed.

**Conclusions:**

Genetically informed precision smoking cessation interventions are feasible in socioeconomically disadvantaged communities, exhibiting high enrollment, engagement, and retention irrespective of race, sex, income, education, or attitudes toward precision smoking treatment. Future smoking cessation interventions in this population should take steps to engage older people and to sustain participation in interventions that include genetic risk counseling.

*Trial registration*: ClinicalTrials.gov No. NCT03521141, Registered 27 April 2018, https://www.clinicaltrials.gov/study/NCT03521141

**Supplementary Information:**

The online version contains supplementary material available at 10.1186/s13722-024-00441-1.

## Background

Smoking is the leading cause of preventable death in the United States, with minority and low-income individuals living in the southern US more likely to develop and die from smoking-related disease [1, 2]. FDA-approved smoking cessation medications (i.e., nicotine replacement, varenicline, bupropion) significantly increase the odds of quitting successfully, yet fewer than one-third of people who are trying to quit smoking use any quit aid, and fewer than 5% use both counseling and medication as recommended by national guidelines [3–5]. This is problematic given that over 95% of unaided quit attempts fail [4, 6–8]. Evidence-based smoking treatment is especially underutilized among Black and low-income individuals, contributing to low quit rates in these populations despite most reporting a desire to quit and many attempting to quit [4, 9–11].

Novel, genetically informed precision approaches to smoking treatment may help engage people who smoke in care and improve outcomes, but the feasibility of implementing these approaches in community settings, especially socioeconomically disadvantaged ones, remains understudied [12–14]. In a survey of predominantly low-income people residing in the southern US who smoke, most (71%) respondents reported favorable attitudes toward precision smoking treatment, but Black race and low education were negative predictors, suggesting potentially greater challenges in reaching these populations [15]. On the other hand, outreach and counseling tailored to specific populations is known to increase the reach and acceptability of smoking cessation interventions [16, 17]. In this respect, the inherently personalized nature of precision interventions has the potential to be a more inclusive antidote to approaches that nominally are “one-size-fits-all” but in fact have been tested in and developed for more advantaged populations.

On balance, whether precision approaches mitigate tobacco-related disparities is likely to be a function of both their overall feasibility in community settings and their ability to reach, engage, and retain in care the more marginalized members of these communities. To address these questions, in a sample of predominantly low-income Black people in the southern US who smoke, the current study examined the feasibility of two genetically informed precision approaches to smoking treatment: (1) counseling and medication selection guided by nicotine metabolism and (2) genetic risk counseling guided by a polygenic risk score for lung cancer.

The rate at which an individual metabolizes nicotine is quantified by the nicotine metabolite ratio (NMR), a validated biomarker that primarily reflects hepatic CYP2A6 activity [18–20]. In a large, multisite randomized controlled trial (RCT) for smoking cessation, fast metabolizers (defined by NMR ≥ 0.31) were approximately twice as likely to quit smoking with varenicline as with nicotine patch [21]. In contrast, among slow metabolizers (NMR < 0.31), varenicline and nicotine patch were equally effective, but varenicline produced more side effects [21]. These results support the general approach of matching fast metabolizers with varenicline and slow metabolizers with nicotine replacement therapy (NRT) [22]. Evidence from a pilot RCT of precision care for smoking cessation among outpatients at an academic medical center found that informing patients of their NMR status improved NMR-medication match rates more than three-fold compared to guideline-based (i.e., non-precision) care [23].

Respiragene^™^ is a commercially available test that calculates a polygenic risk score (PRS) for lung cancer using genetic information along with demographics, smoking history, and personal and family health histories. In previous studies, people who smoke who received Respiragene^™^ PRS results were more likely to undergo lung cancer screening, use NRT, and quit smoking [24, 25]. More broadly, research testing the effects of communicating genetic risk results on smoking behavior suggests a small-to-moderate benefit in cessation rates and, importantly, does not show any negative impact of test results indicating normal or lower genetic risk, such as diminished motivation to quit smoking [26, 27]. Further enthusiasm for this type of precision approach is bolstered by evidence that many people who smoke want to know their genetic susceptibility to smoking-related diseases [15, 28, 29].

In this study, we examined the feasibility of enrolling, engaging, and retaining predominantly low-income Black people in the southern US who smoke in a pilot pragmatic RCT comparing guideline-based care with two precision treatment approaches: metabolism- and PRS-informed care. Objectives were to assess overall trial feasibility, feasibility of each precision intervention, and variation in feasibility with respect to sociodemographic traits and attitudes toward precision smoking treatment. We hypothesized that both the metabolism- and PRS-informed precision interventions would be feasible to implement, that participation would not vary by sociodemographic traits, and that those with more favorable attitudes toward precision smoking treatment would be more likely to enroll, engage, and remain in the study.

## Methods

The Precision Interventions for Smoking (PRISM) trial took place from May 2018 to March 2019 in Tennessee and Mississippi, two southern US states with the 8th- and 5th-highest smoking prevalence in the country, respectively [30]. The trial was administratively based at Vanderbilt University Medical Center (VUMC), an academic medical center in Nashville, Tennessee, but trial activities took place in the community. A timeline of the trial from a participant’s perspective is shown in Additional file 1: Figure S1.

### Population and sample

Participants were recruited through the Southern Community Cohort Study (SCCS), a National Cancer Institute (NCI)-sponsored longitudinal cohort study initiated in 2001 to investigate the determinants of cancer-related health disparities [31]. The cohort includes approximately 85,000 primarily low-income Black adults throughout the southeastern US, along with a large biorepository of blood and saliva samples provided by participants for future research. The study population for the PRISM trial was derived from the Precision Smoking Cessation Survey, which was conducted in 2017 and is described in detail in [15]. This survey sampled SCCS participants in Tennessee and Mississippi who smoked, and yielded 880 respondents. Of these, 458 were excluded due to survey responses that did not meet pre-screening criteria for the PRISM trial (daily smoking, presence of a primary care provider (PCP) or enrollment in Medicare and/or Medicaid (which, under the Affordable Care Act, provides coverage for smoking cessation treatment [32]), and consent to future research contact), and 58 were excluded because they had no blood sample in the SCCS biorepository. In 2018, the remaining 364 individuals were contacted via phone and screened for full eligibility with respect to additional criteria for inclusion (≥ 5 cigarettes smoked per day, medically eligible for and willing to use either nicotine replacement or varenicline, and possession of a valid mailing address and phone number) and exclusion (pregnancy or breastfeeding, cognitive disorder precluding comprehension of study procedures (e.g., dementia), unstable psychiatric disease (suicidal ideation or recent psychiatric hospitalization or medication changes), currently taking smoking cessation medication, or enrolled in another cessation program). Eligible and interested participants initially provided verbal consent by phone and were subsequently required to provide written informed consent to officially enroll in the study.

### Biospecimen processing

After return of written consent, stored blood samples were retrieved from the SCCS biorepository for testing. The NMR and Respiragene™ tests were run on all available specimens regardless of treatment arm.

*Nicotine Metabolite Ratio (NMR).* Blood specimens were analyzed for nicotine, cotinine, and 3-hydroxycotinine (3-HC) via quantitative high-performance liquid chromatography-tandem mass spectrometry at University of Toronto. The NMR was calculated as the ratio of 3-HC to cotinine. Slow metabolizers were defined by NMR < 0.31 and fast metabolizers by NMR ≥ 0.31 [21]. NMR results were deemed invalid (i.e., missing) if the cotinine level was less than 10 ng/ml, indicating that there was no recent nicotine exposure to allow measurement of metabolites [33, 34].

*Respiragene*^*™*^*.* Although typically a saliva test, in this study blood samples were used as they were already available in the SCCS biorepository. Blood samples were extracted for genotyping by the Molecular Epidemiology Core Laboratory of the Institute for Medicine and Public Health at VUMC. The Respiragene™ test estimates a polygenic lung cancer risk score from 1 to 10, with higher numbers indicating higher risk of lung cancer relative to someone who has never smoked. These numeric scores were categorized as “high” (1–4), “higher” (5–7), and “highest” risk (8–10), corresponding to roughly 10, 20, and 40 times higher lung cancer risk, respectively, than someone who has never smoked.

### Study interventions

After return of written consent, participants were contacted by phone for an initial counseling call with a certified tobacco treatment counselor to prepare to quit smoking, during which they were provided with the NCI Clearing the Air program, a booklet to support smoking cessation at any stage of readiness to quit. Participants were then stratified by self-reported cigarettes per day (≥ 10, < 10) and randomized to treatment arm.

The main source of experimental variation in the trial was the *intervention counseling call*, during which a tobacco treatment counselor delivered information and counseling tailored to treatment arm (described further below). Due to the time required to process the biospecimens for the NMR and Respiragene™ tests, the intervention call was scheduled an average of two months after the initial counseling call. Participants were informed that engagement, as defined by completion of the intervention call, was required to continue in the study. Tobacco treatment counselors, study personnel, and participants were not blinded to treatment arm but were blinded to test results outside the treatment arm (e.g., participants assigned to metabolism-informed care received their NMR results but not Respiragene™ results). Upon study completion, all participants were informed of their NMR and Respiragene™ test results and given the opportunity to discuss them with a tobacco treatment counselor.

*Guideline-Based Care (GBC).* In the GBC arm, during the intervention counseling call, participants and tobacco treatment counselors discussed FDA-approved smoking cessation medications (i.e., varenicline and NRT) and then co-selected one through shared decision-making. Participants were also offered a referral to the state quitline. Following the call, to facilitate a medication prescription, study personnel sent a fax to participants’ PCPs stating their patients’ chosen medication and preferred pharmacy, with a request to both prescribe the medication for the patient and fax a copy of the prescription to the study team for verification.

At 2 weeks and 2 months after the intervention call, respectively, tobacco treatment counselors conducted follow-up calls to identify and troubleshoot problems with obtaining study medication or taking it once obtained. These medication check-ins were designed to support guideline-based use of both counseling and pharmacotherapy and did not involve data collection intended for analysis.

*GBC plus nicotine metabolism-informed care (MIC).* Participants in the MIC arm received all components of GBC with one modification: shared decision-making during the intervention call regarding medication selection was guided by the participant’s NMR status (i.e., varenicline for fast metabolizers and NRT for slow metabolizers). To aid discussion of NMR results, the tobacco treatment counselor used an infographic developed by the study team with input from a community advisory board [35] (Additional file 1: Figure S2). The NMR results and infographic were mailed to study participants in advance of the intervention call to facilitate discussion. These materials were also sent to the participant’s PCP. Participants whose NMR test did not yield a valid result were informed that their blood sample likely did not have enough nicotine exposure at the time of their blood draw, and counselors provided a general, rather than individually tailored, description of the NMR and its implications for medication selection. Though medication selection in this arm was guided by NMR test results, patients’ choices and providers’ prescriptions could result in patients receiving medications discordant with their NMR status.

*GBC plus lung cancer polygenic risk score counseling (PRS).* Participants in the PRS arm received all components of GBC with one modification: tobacco treatment counselors provided counseling related to the participant’s Respiragene™ polygenic risk score during the intervention call. To aid discussion of the PRS, the tobacco treatment counselor used an infographic developed by the study team with input from a community advisory board (Additional file 1: Figure S2) [35]. The genetic test result and infographic were mailed to study participants in advance of the intervention call to facilitate discussion. These materials were also sent to the participant’s PCP. Participants’ PRS scores did not explicitly influence medication selection or prescription, though this may have occurred organically (e.g., higher-risk participants, upon learning their PRS score, may have developed a stronger preference for varenicline, or PCPs may have been more likely to prescribe medication for these patients).

### Data collection, outcome measures, and analytic methods

*Participant characteristics and trial outcomes.* Baseline sociodemographic variables including age, sex, race, highest education completed, and annual household income were gathered during prior survey waves of the SCCS, including the Precision Smoking Cessation Survey conducted one year prior to the launch of the pilot RCT. Sex (male vs. female), race (Black vs. non-Black), highest education completed (high school diploma and beyond vs. no high school diploma), and annual household income ($15,000 and above vs. less than $15,000) were operationalized as binary variables. Age was treated as a continuous variable. Other baseline variables were collected through a survey conducted at the time of study enrollment.

The Precision Smoking Cessation Survey also collected two self-report measures of attitude toward precision smoking treatment, in which participants were asked, “If a blood test could help my doctor choose the best medicine for me to quit smoking, I would take that blood test” (henceforth “attitude toward NMR testing”), and “If a saliva test could use information on my genes to predict my risk of getting lung cancer, I would take the saliva test” (henceforth “attitude toward PRS testing”). Both were queried with Likert scales ranging from 1 (“strongly disagree”) to 5 (“strongly agree”). A “favorable” attitude was defined as a response of 4 (“agree”) or 5.

Collection of outcomes data, which was restricted to those completing the intervention counseling call, occurred through phone surveys at 1, 3, and 6 months after the intervention call. At each follow-up survey, participants were asked about their smoking status and if they had received their chosen smoking cessation medication from any source. At 6-month follow-up, for participants reporting 7-day point prevalence smoking abstinence, saliva samples were requested through mail using kits manufactured by Salimetrics, LLC. Biochemical verification of self-reported abstinence was assessed through salivary cotinine testing (cotinine ≤ 10 ng/ml) performed at the VUMC Pathology Laboratory.

*Feasibility measures.* Feasibility was assessed by rates of study enrollment (proportion of eligible individuals enrolling in the study), intervention engagement (proportion of enrolled participants completing the intervention counseling call), and retention (proportion of engaged participants completing the 6-month follow-up survey). We also examined two secondary measures of engagement defined as (1) the proportion of enrolled participants both completing the intervention call and receiving their chosen smoking cessation medication during the course of the study and (2) the proportion of participants completing the intervention call (denominator) receiving their chosen smoking cessation medication during the course of the study (numerator). This level of “full” engagement with respect to both counseling and pharmacotherapy was deemed clinically important, as treatment with both modalities was a focus of the intervention call and is more effective than either alone and hence guideline-recommended [3].

The target rate to establish feasibility was > 70% for each stage of implementation. Rates were calculated as binomial proportions, with 95% confidence intervals estimated using the Wilson score method [36].

*Differential enrollment, engagement, and retention.* Variation in feasibility was assessed by testing for selection effects at each stage of implementation. We conducted logistic regressions of each implementation stage (enrollment, engagement, retention) on age, sex, race, income, education, and attitudes toward NMR and PRS testing. At each stage, the sample was restricted to those “at-risk” (e.g., retention among engaged participants only, not all eligible participants) in order to isolate selection effects at each stage rather than cumulatively. Robust standard errors were estimated throughout. All analysis was conducted using Stata version 17.

## Results

### Sample characteristics

Table 1 summarizes baseline demographics, smoking history and attitudes, biosample measurements, and treatment assignment of five subsamples corresponding to progressive stages of study implementation. The *eligible* population was predominantly elderly (mean age 60 years), female (66.3%), Black (79.3%), high school-graduated (72.7%), and low-income (68.2% with household income less than $15,000 per year). These raw proportions were qualitatively unchanged among those enrolling in the study, those engaging in the study interventions, and those remaining in the study through 6-month follow-up. Results also indicate that most (75.0% and 78.7%) eligible participants had favorable attitudes toward the NMR and PRS tests, respectively, and this proportion was roughly similar across the stages of implementation. In the eligible population, of the demographic variables presented in Table 1, only education was significantly associated with either attitude: the odds of a favorable attitude toward the NMR test was 3.3 times higher among those with a high school diploma vs. those without (p = 0.031; not shown).

**Table 1:** Sample characteristics, by trial stage

	Eligible (n = 92)	Enrolled (n = 67)	Engaged		Retained (n = 43)
			All (n = 58)	Received med (n = 43)	
	(1)	(2)	(3a)	(3b)	(4)
*Demographics*					
Age (years)	60.0	59.7	59.2	58.8	58.9
Female sex (%)	66.3	70.1	72.4	72.1	72.1
Black race (%)	79.3	79.1	81.0	76.7	81.4
High school graduate (%)	72.7	75.0	78.2	78.0	78.0
Household income < $15,000 (%)	68.2	65.1	63.6	60.0	63.4
*Smoking history and attitudes*					
Cigarettes per day	13.4	13.8	13.5	14.8	13.7
Age started smoking (years)	17.6	17.3	17.4	17.1	16.7
Time to first cigarette ≤ 30 min (%)	68.5	68.7	69.0	74.4	62.8
Planning to quit smoking (%)	63.5	63.0	64.1	64.5	58.6
Confidence in quitting (1–5)	3.26	3.23	3.27	3.19	3.27
Favorable attitude toward NMR test (%)	75.0	77.8	77.8	78.0	77.5
Favorable attitude toward PRS test (%)	78.7	78.5	75.0	74.4	71.4
*Biosample measurements*					
Nicotine metabolite ratio (3-HC/cotinine)		0.410	0.386	0.406	0.383
Fast metabolizer (%)		56.7	53.5	58.1	53.5
Slow metabolizer (%)		35.8	37.9	30.2	39.5
Invalid result (%)		7.5	8.6	11.6	7.0
Respiragene™ polygenic risk score (1–10)		5.78	5.57	5.53	5.67
High risk (%)		38.8	39.7	41.9	37.2
Higher risk (%)		31.3	34.5	34.9	34.9
Highest risk (%)		29.9	25.9	23.3	27.9

All values are means. “Engaged” refers to completion of the intervention counseling call; Column 3a comprises all participants who completed the intervention call, whereas Column 3b comprises all participants who both completed the intervention call and reported receiving their chosen smoking cessation medication during the study. “Retained” refers to completion of the 6-month follow-up survey. “Fast” and “slow” metabolizers correspond to NMR ≥ 0.31 and NMR < 0.31, respectively. “High,” “higher,” and “highest” risk correspond to polygenic risk scores of 1–4, 5–7, and 8–10, respectively

*NMR* nicotine metabolite ratio, *PRS* polygenic risk score, *3-HC* 3-hydroxycotinine

As described above, biosample measurements were obtained for the 67 enrolled participants only. Among these, 38 (56.7%) were classified as fast metabolizers, 24 (35.8%) were classified as slow metabolizers, and 5 (7.5%) could not be classified due to invalid NMR results. Lung cancer risk as predicted by the Respiragene^™^ PRS was distributed roughly uniformly across the three risk categories: 26 (38.8%) were high-risk, 21 (31.3%) were higher-risk, and 20 (29.9%) were highest-risk. There were no invalid results for the PRS.

### Feasibility

**Fig. 1:**
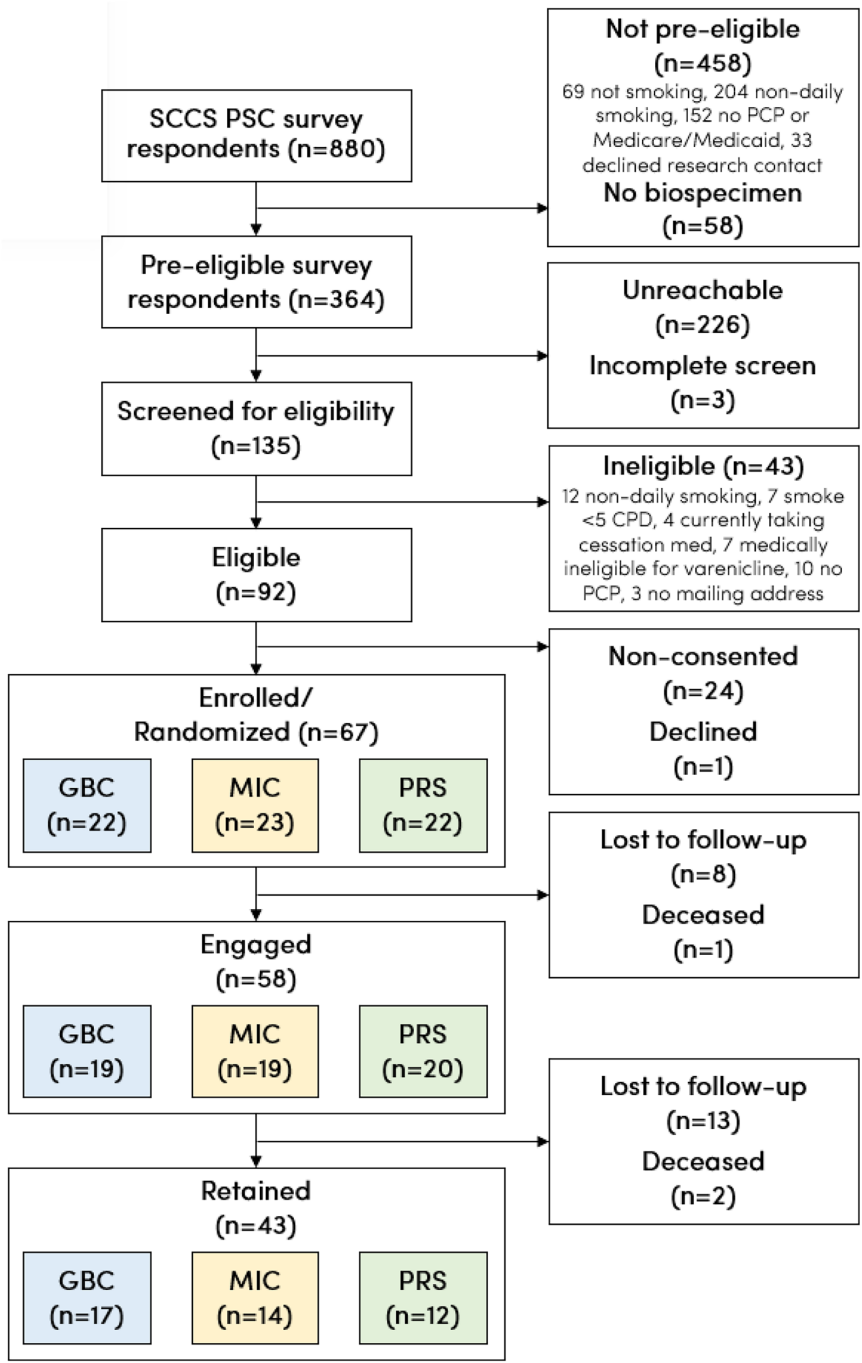
Flow of participants in pilot RCT. “Pre-eligible” refers to meeting eligibility criteria based on responses to the PSC survey conducted before the launch of the pilot RCT. “Engaged” refers to completion of the intervention counseling call. “Retained” refers to completion of the 6-month follow-up survey. *SCCS* Southern Community Cohort Study, *PSC* Precision Smoking Cessation, *CPD* cigarettes per day, *PCP* primary care provider, *GBC* Guideline-Based Care, *MIC* Metabolism-Informed Care, *PRS* Polygenic Risk Score

Table 2 reports rates of enrollment, engagement, and retention both overall and by treatment arm, and Figure 1 details the reasons for attrition at each stage. Of the 364 SCCS participants meeting pre-screening criteria, 226 (62.1%) could not be reached for eligibility screening, three (0.8%) did not complete eligibility screening, and 43 (11.8%) completed screening but did not meet eligibility criteria. Of the remaining 92 (25.3%) participants confirmed to be eligible, 24 (26.1%) verbally consented but did not return written consent as required by the institutional review board, and one (1.1%) declined participation. Thus, of 92 eligible participants, 67 (72.8%; 95% confidence interval (CI) 63.0–80.9%) enrolled and were randomized.

**Table 2:** Rates of study enrollment, engagement, and retention, overall and by treatment arm

Eligible N = 92	Enrolled/Randomized	Engaged		Retained
		All	Received Med	
	(1)	(2a)	(2b)	(3)
*Full sample (N)*	67	58	43	43
Marginal rate (% of prior stage)	72.8 [63.0, 80.9]	86.6 [76.4, 92.8]	64.2 [52.2, 74.6]	74.1 [61.6, 83.7]
Cumulative rate (% of eligible)	72.8 [63.0, 80.9]	63.0 [52.8, 72.2]	46.7 [36.9, 56.9]	46.7 [36.9, 56.9]
*MIC arm only (N)*	23	19	11	14
Marginal rate (% of prior stage)		82.6 [62.9, 93.0]	47.8 [29.2, 67.0]	73.7 [51.2, 88.2]
Odds ratio (MIC vs. GBC)		0.75 [0.15, 3.86]	0.34 [0.10, 1.20]	0.33 [0.05, 2.00]
*PRS arm only (N)*	22	20	16	12
Marginal rate (% of prior stage)		90.9 [72.2, 97.5]	72.7 [51.8, 86.8]	60.0 [38.7, 78.1]
Odds ratio (PRS vs. GBC)		1.58 [0.23, 10.67]	1.00 [0.26, 3.81]	0.18* [0.03, 1.00]

The top panel (“Full Sample”) reports marginal and cumulative rates for each stage of study implementation, where “marginal” denotes rates as a proportion of the prior stage. The middle (“MIC arm only”) and bottom (“PRS arm only”) panels show treatment arm-specific marginal rates as well as results of a logistic regression of each stage (column header) on dummy variables for each of the two precision treatment arms (reference group: Guideline-Based Care arm). “Engaged” refers to completion of the intervention counseling call; Column 2a comprises all participants who completed the intervention call, whereas Column 2b comprises all participants who both completed the intervention call and reported receiving their chosen smoking cessation medication during the study. “Retained” refers to completion of the 6-month follow-up survey

*MIC* Metabolism-Informed Care, *PRS* Polygenic Risk Score

^*^Significant at the 5% level

Of the 67 enrolled participants, 58 (86.6%; 95% CI 76.4–92.8%) completed the intervention counseling call in which counseling and medication selection were customized by treatment arm. Furthermore, Table 2, Column 2b shows that 64.2% (95% CI 52.2–74.6%) of enrolled participants both completed the intervention call and reported receiving their chosen smoking cessation medication over the 6-month follow-up period of the study. As a proportion of engaged participants, this figure was 74.1% (i.e., 43 of 58 participants completing the intervention call reported receiving study medication; 95% CI 61.6–83.7%; not shown). Finally, of the 58 engaged participants, 43 (74.1%; 95% CI 61.6–83.7%) were retained at 6-month follow-up. Cumulatively, 46.7% (95% CI 36.9–56.9%) of eligible participants enrolled in the study, engaged with the intervention, and completed the 6-month follow-up survey. The study completion rate among enrolled participants, a more standard measure of cumulative retention, was 64.2% (43 of 67 participants; 95% CI 52.2–74.6%; not shown).

The middle and bottom panels of Table 2 report rates of engagement and retention by treatment arm (rates of enrollment by treatment arm are not applicable since enrollment occurred before randomization). In addition, each column reports the odds ratios (OR) of a logistic regression of each implementation stage on the two treatment arms, which formally tests the hypothesis of whether engagement and retention in each precision treatment arm differed from those in GBC. Results show that engagement in each precision treatment arm did not significantly differ from that in GBC (Columns 2a and 2b), although there was a trend toward participants randomized to MIC having lower odds of receiving their chosen smoking cessation medication during the study (OR 0.34, 95% CI 0.10–1.20, p = 0.095). Regarding retention, conditional on engagement, randomization to PRS (vs. GBC) was associated with marginally significantly lower odds of retention at 6 months (OR 0.18, 95% CI 0.03–1.00, p = 0.050).

### Differential enrollment, engagement, and retention

Table 3 examines whether feasibility of enrollment, engagement, and retention varied with respect to demographic traits and attitudes toward precision smoking treatment. Each column represents a logistic regression of an implementation stage (column header) on a set of explanatory variables, with odd-numbered columns examining demographic traits and even-numbered columns examining attitudes. Columns 1 and 2 show that neither demographics nor attitudes toward the NMR and PRS tests predicted eligible respondents’ decision to enroll in the study. In contrast, Column 3 shows that, conditional on enrollment and controlling for other demographic traits, older age was associated with lower odds of engagement as defined by completion of the intervention counseling call (OR 0.83, 95% CI 0.73–0.95, p = 0.008). There was no significant association between attitudes toward precision smoking treatment and intervention engagement. Finally, Columns 7 and 8 show that neither demographic traits nor attitudes toward precision smoking treatment influenced retention through 6-month follow-up.

**Table 3:** Selection effects with respect to demographic traits and attitudes toward precision smoking treatment

	Enrolled (n = 92)		Engaged				Retained (n = 43)	
			All (n = 58)		Received med (n = 43)			
	(1)	(2)	(3)	(4)	(5)	(6)	(7)	(8)
Demographics								
Age	0.99 [0.91, 1.07]		0.83* [0.73, 0.95]		0.93 [0.85, 1.08]		1.00 [0.91, 1.10]	
Female sex	1.80 [0.66, 4.93]		2.44 [0.53, 11.23]		1.35 [0.39, 4.62]		0.79 [0.18, 3.36]	
Black race	0.94 [0.29, 3.03]		3.44 [0.65, 18.20]		0.55 [0.14, 2.06]		1.18 [0.26, 5.32]	
High school graduate	1.15 [0.39, 3.41]		3.88 [0.69, 21.92]		1.54 [0.46, 5.22]		1.02 [0.24, 4.41]	
Household income < $15,000	0.61 [0.19, 2.00]		0.68 [0.10, 4.52]		0.56 [0.17, 1.79]		1.24 [0.36, 4.31]	
Favorable attitude toward:								
Nicotine metabolite ratio test		1.54 [0.51, 4.68]		1.71 [0.28, 10.49]		1.06 [0.25, 4.44]		1.28 [0.23, 7.20]
Polygenic risk score test		1.02 [0.31, 3.42]		1 (omitted)		0.37 [0.06, 2.31]		0.51 [0.07, 3.83]
p-value of model chi-squared test	0.803	0.725	0.126	0.560	0.503	0.485	0.999	0.801
Pseudo-R-squared	0.026	0.006	0.236	0.007	0.055	0.020	0.005	0.010

Each column reports adjusted odds ratios [95% confidence intervals] of a logistic regression of each stage of implementation (column header) on a set of explanatory variables (rows). “Engaged” refers to completion of the intervention counseling call; Columns 3 and 4 each comprise all participants who completed the intervention call, whereas Columns 5 and 6 each comprise all participants who both completed the intervention call and reported receiving their chosen smoking cessation medication during the study. “Retained” refers to completion of the 6-month follow-up survey

^*^Significant at the 5% level

## Discussion

In this pilot feasibility trial of precision smoking cessation interventions among a predominantly Black, low-income community sample of adults who smoke, 72.8% of eligible individuals enrolled in the study, 86.6% of enrolled participants engaged with the intervention, and 74.1% of engaged participants were retained through six months of follow-up, exceeding targets for feasibility of enrollment, engagement, and retention. Furthermore, examination of selection effects revealed that enrollment, engagement, and retention did not vary by sex, race, education, income, or attitudes toward precision smoking treatment. This finding helps allay concerns about precision approaches inadvertently excluding some groups, such as those who are socioeconomically more disadvantaged or who report less favorable attitudes toward precision approaches that require the collection of biological specimens, and appears to support broad representativeness of reach.

Taken together, this study provides preliminary support for the feasibility of implementing precision smoking interventions in the low-income community setting in an equitable manner. A previous survey of predominantly low-income Black SCCS participants who smoke found that most had favorable attitudes toward precision approaches to smoking cessation treatment [15]. The current experimental findings build on this research, with high rates of study enrollment, engagement, and retention providing behavioral confirmation of self-reported attitudes.

Despite overall supportive findings, results also highlight several potential challenges of implementing precision approaches in the low-income community setting. First, while enrollment rates were high among confirmed-eligible individuals, many pre-screened individuals could not be reached via phone for formal eligibility screening. Future interventions may be able to reach more community members who smoke by collaborating with local healthcare and community institutions.

Second, medication use was lower than expected. Although the rate of combined use of counseling and medication (64.2% of enrolled participants) far exceeded the roughly 5% national average in the general adult smoking population [4], nevertheless, 15 of 58 (25.9%) participants who completed the intervention counseling call did not receive their chosen smoking cessation medication during the study. This barrier may have been due in part to lack of engagement from participants’ PCPs, who were responsible for prescribing smoking cessation medications for their patients. Future research should focus on enhancing community providers’ engagement in precision smoking treatment by taking more active approaches, such as embedding interventions within their clinics.

Third, conditional on enrollment in the trial, older age was associated with lower engagement in the intervention counseling call (OR 0.83, 95% CI 0.73–0.95, p = 0.008). While this finding warrants qualitative probing as well as confirmation in larger studies, it raises the possibility that older people who smoke may have lower willingness and/or ability to participate in longitudinal phone counseling as part of precision smoking cessation interventions. Regardless, given that age is a strong predictor of smoking-related disease and death, it is important to ensure that precision smoking cessation interventions do not exclude high-risk individuals on account of their older age.

Finally, conditional on engagement, retention was significantly lower in the PRS arm than in the GBC arm (OR 0.18, 95% CI 0.03–1.00, p = 0.050). While this marginal result may have been due to random chance in a small sample, it is also possible that delivering PRS results to this population may have been associated with unintended effects, such as fatalism among those at higher risk of lung cancer or complacency among those at lower risk [37]. Future research in socioeconomically disadvantaged community settings should focus on strategies to maximize understanding of genetic risk and its implications for personal success in quitting—a task that is likely to require participatory research to develop counseling messages and tools tailored to this population [35, 38].

This study has several methodological limitations. First, the sample size was relatively small, with only 92 eligible participants and 67 enrolled. This may have limited the statistical power to detect differences in enrollment, engagement, and retention by treatment arm and by participant traits and attitudes. For example, Black participants had 3 times the odds of engaging with the study than White participants (Table 3, Column 3), but this point estimate was not statistically significant due to the small sample size. Second, the study selected for individuals who had already contributed biospecimens to the SCCS biorepository; it therefore may overstate the acceptability of precision smoking interventions in the general smoking population, which may be less willing to provide biospecimens, though the fact that those with unfavorable attitudes toward precision smoking treatment were no less likely to participate in the study is reassuring. Third, even among those willing to provide biospecimens, the reliance of the NMR on recent nicotine exposure for valid measurement meant that some specimens did not yield actionable results, as was the case for 7.5% of the specimens in this study. Fourth, there was a lack of robust measurement of provider engagement in participants’ quit attempts and, specifically, provision of prescriptions for smoking cessation medication. Thus, we can only speculate regarding the extent to which participant vs. provider factors influenced participants’ access to and use of cessation medication. Finally, although efficacy outcomes such as the proportion of participants whose medications matched their NMR status and the proportion abstaining from smoking were measured, because of small sample sizes, we were not able to formally test whether these outcomes varied by treatment arm.

Despite these limitations, the study has several methodological strengths, with significant implications for future research and clinical practice. This study is the first to test the feasibility of delivering, in any community setting, interventions based on the NMR, a genetically informed biomarker of nicotine metabolism with the potential to increase medication efficacy and decrease side effects, and on the Respiragene™ PRS, a gene-based lung cancer risk score which may enhance commitment to behavior change among people who smoke. Community roll-out of these interventions will be important for maximizing their real-world effectiveness. Moreover, the study population consists of people who smoke from social groups that are traditionally underrepresented in healthcare research and are disproportionately burdened by tobacco. Understanding their likelihood of enrollment, engagement, and retention in precision smoking treatment research lays the foundation for larger intervention studies to more formally examine the equity of these approaches [39, 40]. This work also establishes the utility of the SCCS biorepository and the more general approach of collecting and storing biospecimens to facilitate genetically guided interventions such as the NMR and Respiragene™ PRS. Finally, this study directly links and compares attitudes and actions regarding precision smoking treatment—i.e., whether attitudes toward precision smoking treatment affect actual decisions to participate in such treatment. We show that, using counseling messages developed with community input, precision smoking cessation interventions can recruit, engage, and retain in care even those with unfavorable attitudes toward precision smoking treatment.

## Conclusions

Precision approaches to smoking cessation that account for the genetics of smoking have the potential to improve quit rates and mitigate racial and socioeconomic disparities in smoking-related disease and death. However, whether such approaches are feasible in low-income, predominantly minority community settings is unknown. This study’s findings suggest that precision smoking treatment can be feasibly studied and implemented in community settings among groups that are at especially high risk of tobacco-related disease and mortality. These results lay the groundwork for future studies examining the comparative effectiveness of precision approaches to smoking cessation treatment in community settings, as well as studies evaluating strategies to ensure the equitable implementation of these approaches at scale.

## Supplementary Information

**Additional file 1: Figure S1.** Study timeline by stage of implementation. NMR: nicotine metabolite ratio; PRS: polygenic risk score; PCP: primary care provider. **Figure S2.** Sample infographics mailed to participants in the MIC (left) and PRS (right) arms of pilot RCT. Infographics were co-developed with a community advisory board that included people who smoke as well as do not smoke, as detailed in [35]. MIC: Metabolism-Informed Care; PRS: Polygenic Risk Score.

## Data Availability

Data may be available upon request, pending approval of the study steering committee. Statistical code is available upon request.

## Abbreviations

3-HC: 3-Hydroxycotinine
FDA: Food and Drug Administration
GBC: Guideline-based care
MIC: Metabolism-informed care
NCI: National Cancer Institute
NMR: Nicotine metabolite ratio
NRT: Nicotine replacement therapy
PCP: Primary care provider
PRISM: Precision Interventions for Smoking
PRS: Polygenic risk score
RCT: Randomized controlled trial
SCCS: Southern Community Cohort Study
VUMC: Vanderbilt University Medical Center

## References

[1] Singh GK, et al. Socioeconomic, Rural-Urban, and racial inequalities in US cancer mortality: part i—all cancers and lung cancer and part ii—colorectal, prostate, breast, and cervical cancers. J Cancer Epidemiol. 2011. 10.1155/2011/107497. 22496688 10.1155/2011/107497 PMC3307012

[2] Health, U.S.D.o. and S. Human. The Health Consequences of Smoking: 50 Years of Progress. a report of the surgeon general. 2014, Atlanta (GA).

[3] Fiore MC, et al. Treating tobacco use and dependence: 2008 update. Rockville, MD: US Department of Health and Human Services; 2008.

[4] Babb S. Quitting smoking among adults—United States, 2000–2015. MMWR Morb Mortal Wkl Rep. 2017;65:1457. 10.15585/mmwr.mm6552a1 28056007

[5] Salloum RG, et al. Smoking-cessation methods and outcomes among cancer survivors. Am J Prev Med. 2020;59(4):615–7. 32446750 10.1016/j.amepre.2020.03.016

[6] Hughes JR, Keely J, Naud S. Shape of the relapse curve and long-term abstinence among untreated smokers. Addiction. 2004;99(1):29–38. 14678060 10.1111/j.1360-0443.2004.00540.x

[7] Kasza KA, et al. Effectiveness of stop-smoking medications: findings from the International Tobacco Control (ITC) Four Country Survey. Addiction. 2013;108(1):193–202. 22891869 10.1111/j.1360-0443.2012.04009.x PMC3500450

[8] Kotz D, Brown J, West R. ‘Real-world’ effectiveness of smoking cessation treatments: a population study. Addiction. 2014;109(3):491–9. 24372901 10.1111/add.12429

[9] Cokkinides VE, et al. Racial and ethnic disparities in smoking-cessation interventions: analysis of the 2005 National Health interview survey. Am J Prev Med. 2008;34(5):404–12. 18407007 10.1016/j.amepre.2008.02.003

[10] Pacek LR, McClernon FJ, Bosworth HB. Adherence to pharmacological smoking cessation interventions: a literature review and synthesis of correlates and barriers. Nicotine Tob Res. 2018;20(10):1163–72. 29059394 10.1093/ntr/ntx210 PMC6121917

[11] Trinidad DR, et al. A nationwide analysis of US racial/ethnic disparities in smoking behaviors, smoking cessation, and cessation-related factors. Am J Public Health. 2011;101(4):699–706. 21330593 10.2105/AJPH.2010.191668 PMC3052356

[12] Bourdon JL, et al. In-vivo design feedback and perceived utility of a genetically-informed smoking risk tool among current smokers in the community. BMC Med Genomics. 2021;14(1):139. 34039360 10.1186/s12920-021-00976-1 PMC8152342

[13] Carroll DM, et al. Exploring potential for a personalized medicine approach to smoking cessation with an American Indian tribe. Nicotine Tob Res. 2022;25(1):120–6. 10.1093/ntr/ntac141 PMC9717394 35661899

[14] Chen L-S, et al. Genomic medicine to reduce tobacco and related disorders: translation to precision prevention and treatment. Addict Neurosci. 2023;7:100083. 37602286 10.1016/j.addicn.2023.100083 PMC10434839

[15] Senft N, et al. Attitudes towards precision treatment of smoking in the Southern Community Cohort Study. Cancer Epidemiol Biomarkers Prev. 2019. 10.1158/1055-9965.EPI-19-0179. 31160346 10.1158/1055-9965.EPI-19-0179 PMC6679740

[16] Matthews AK, Sánchez-Johnsen L, King A. Development of a culturally targeted smoking cessation intervention for African American smokers. J Community Health. 2009;34(6):480–92. 19728056 10.1007/s10900-009-9181-5 PMC3712791

[17] D’Angelo H, et al. Achieving equity in the reach of smoking cessation services within the NCI cancer Moonshot-funded cancer center cessation initiative. Health Equity. 2021;5(1):424–30. 34235367 10.1089/heq.2020.0157 PMC8237098

[18] Dempsey D, et al. Nicotine metabolite ratio as an index of cytochrome P450 2A6 metabolic activity. Clin Pharmacol Ther. 2004;76(1):64–72. 15229465 10.1016/j.clpt.2004.02.011

[19] Schnoll RA, et al. Nicotine metabolic rate predicts successful smoking cessation with transdermal nicotine: a validation study. Pharmacol Biochem Behav. 2009;92(1):6–11. 19000709 10.1016/j.pbb.2008.10.016 PMC2657225

[20] West O, Hajek P, McRobbie H. Systematic review of the relationship between the 3-hydroxycotinine/cotinine ratio and cigarette dependence. Psychopharmacology. 2011;218(2):313–22. 21597990 10.1007/s00213-011-2341-1

[21] Lerman C, et al. Use of the nicotine metabolite ratio as a genetically informed biomarker of response to nicotine patch or varenicline for smoking cessation: a randomised, double-blind placebo-controlled trial. Lancet Respir Med. 2015;3(2):131–8. 25588294 10.1016/S2213-2600(14)70294-2 PMC4480925

[22] Siegel SD, et al. The use of biomarkers to guide precision treatment for tobacco use. Addict Neurosci. 2023;6:100076. 37089247 10.1016/j.addicn.2023.100076 PMC10121195

[23] Wells QS, et al. Nicotine metabolism-informed care for smoking cessation: a pilot precision RCT. Nicotine Tob Res. 2017. 10.1093/ntr/ntx235. 10.1093/ntr/ntx235 PMC6236077 29059367

[24] de Viron S, et al. Impact of genetic notification on smoking cessation: systematic review and pooled-analysis. PLoS ONE. 2012;7(7):e40230. 22808123 10.1371/journal.pone.0040230 PMC3394798

[25] Young RP, Hopkins RJ, Gamble GD. Clinical applications of gene-based risk prediction for lung cancer and the central role of chronic obstructive pulmonary disease. Front Genet. 2012;3:210. 23087706 10.3389/fgene.2012.00210 PMC3472507

[26] Frieser MJ, Wilson S, Vrieze S. Behavioral impact of return of genetic test results for complex disease: systematic review and meta-analysis. Health Psychol. 2018;37(12):1134–44. 30307272 10.1037/hea0000683 PMC6263735

[27] Hollands GJ, et al. The impact of communicating genetic risks of disease on risk-reducing health behaviour: systematic review with meta-analysis. BMJ. 2016;352:i1102. 26979548 10.1136/bmj.i1102 PMC4793156

[28] Olfson E, et al. Implications of personal genomic testing for health behaviors: the case of smoking. Nicotine Tob Res. 2016;18(12):2273–7. 27613923 10.1093/ntr/ntw168 PMC5103936

[29] Smerecnik C, Grispen JEJ, Quaak M. Effectiveness of testing for genetic susceptibility to smoking-related diseases on smoking cessation outcomes: a systematic review and meta-analysis. Tob Control. 2012;21(3):347–54. 21948804 10.1136/tc.2011.042739

[30] Centers for Disease Control and Prevention State Tobacco Activities Tracking & Evaluation (STATE) System. Map of Current Cigarette Use Among Adults (Behavioral Risk Surveillance System) 2019.

[31] Signorello LB, Hargreaves MK, Blot WJ. The Southern Community Cohort Study: investigating health disparities. J Health Care Poor Underserved. 2010;21(1 Suppl):26–37. 20173283 10.1353/hpu.0.0245 PMC2940058

[32] McAfee T, et al. Helping smokers quit — opportunities created by the affordable care act. N Engl J Med. 2015;372(1):5–7. 25409263 10.1056/NEJMp1411437 PMC4465216

[33] Vartiainen E, et al. Validation of self reported smoking by serum cotinine measurement in a community-based study. J Epidemiol Community Health. 2002;56(3):167–70. 11854334 10.1136/jech.56.3.167 PMC1732104

[34] Giratallah HK, et al. Nicotine metabolite ratio: comparison of the three urinary versions to the plasma version and nicotine clearance in three clinical studies. Drug Alcohol Depend. 2021;223:108708. 33873029 10.1016/j.drugalcdep.2021.108708 PMC8133391

[35] Connors E, et al. Improving community advisory board engagement in precision medicine research to reduce health disparities. J Health Dispar Res Pract. 2020;12(6):80. PMC7442965 32832256

[36] Newcombe R. Two-sided confidence intervals for the single proportion: comparison of seven methods. Stat Med. 1998;17:857–72. 9595616 10.1002/(sici)1097-0258(19980430)17:8<857::aid-sim777>3.0.co;2-e

[37] Senft Everson N, et al. Dispositional optimism and optimistic bias: associations with cessation motivation, confidence, and attitudes. Health Psychol. 2022;41(9):621–9. 35901400 10.1037/hea0001184 PMC9830640

[38] Ramsey AT, et al. Participatory design of a personalized genetic risk tool to promote behavioral health. Cancer Prev Res (Phila). 2020;13(7):583–92. 32209550 10.1158/1940-6207.CAPR-20-0029 PMC7335332

[39] Gray M, Lagerberg T, Dombrádi V. Equity and value in “precision medicine.” New Bioeth. 2017;23(1):87–94. 28517992 10.1080/20502877.2017.1314891

[40] Matthew DB. Two threats to precision medicine equity. Ethn Dis. 2019;29(Suppl 3):629–40. 31889768 10.18865/ed.29.S3.629 PMC6919972

