# Supplementary material for: Feasibility of precision smoking treatment in a low-income community setting: results of a pilot randomized controlled trial in The Southern Community Cohort Study
Source: Addict Sci Clin Pract. 2024 Mar 15;19:16. doi: 10.1186/s13722-024-00441-1 (PMC10941447; doi:10.1186/s13722-024-00441-1)
Supplement: Supplementary file 1 — Additional file 1: Figure S1. Study timeline by stage of implementation. NMR: nicotine metabolite ratio; PRS: polygenic risk score; PCP: primary care provider. Figure S2. Sample infographics mailed to participants in the MIC (left) and PRS (right) arms of pilot RCT. Infographics were co-developed with a community advisory board that included people who smoke as well as do not smoke, as detailed in [35]. MIC: Metabolism-Informed Care; PRS: Polygenic Risk Score. [file 13722_2024_441_MOESM1_ESM.docx]

**Additional Figure 1. Study timeline by stage of implementation.**


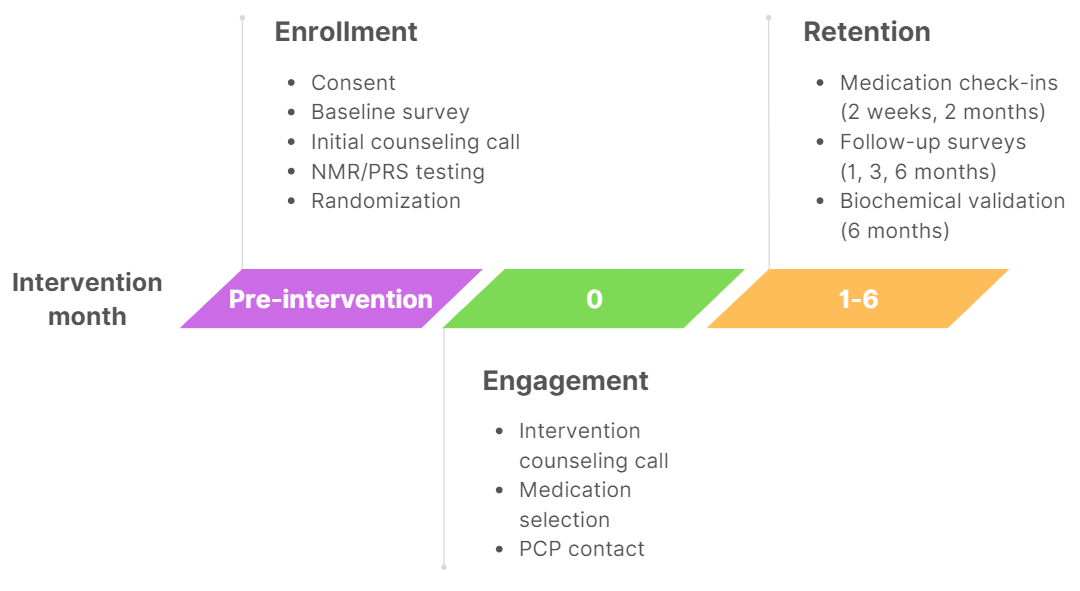


*Note:* NMR: nicotine metabolite ratio; PRS: polygenic risk score; PCP: primary care provider.

**Additional Figure 2. Sample infographics mailed to participants in the MIC (left) and PRS (right) arms of pilot RCT.**


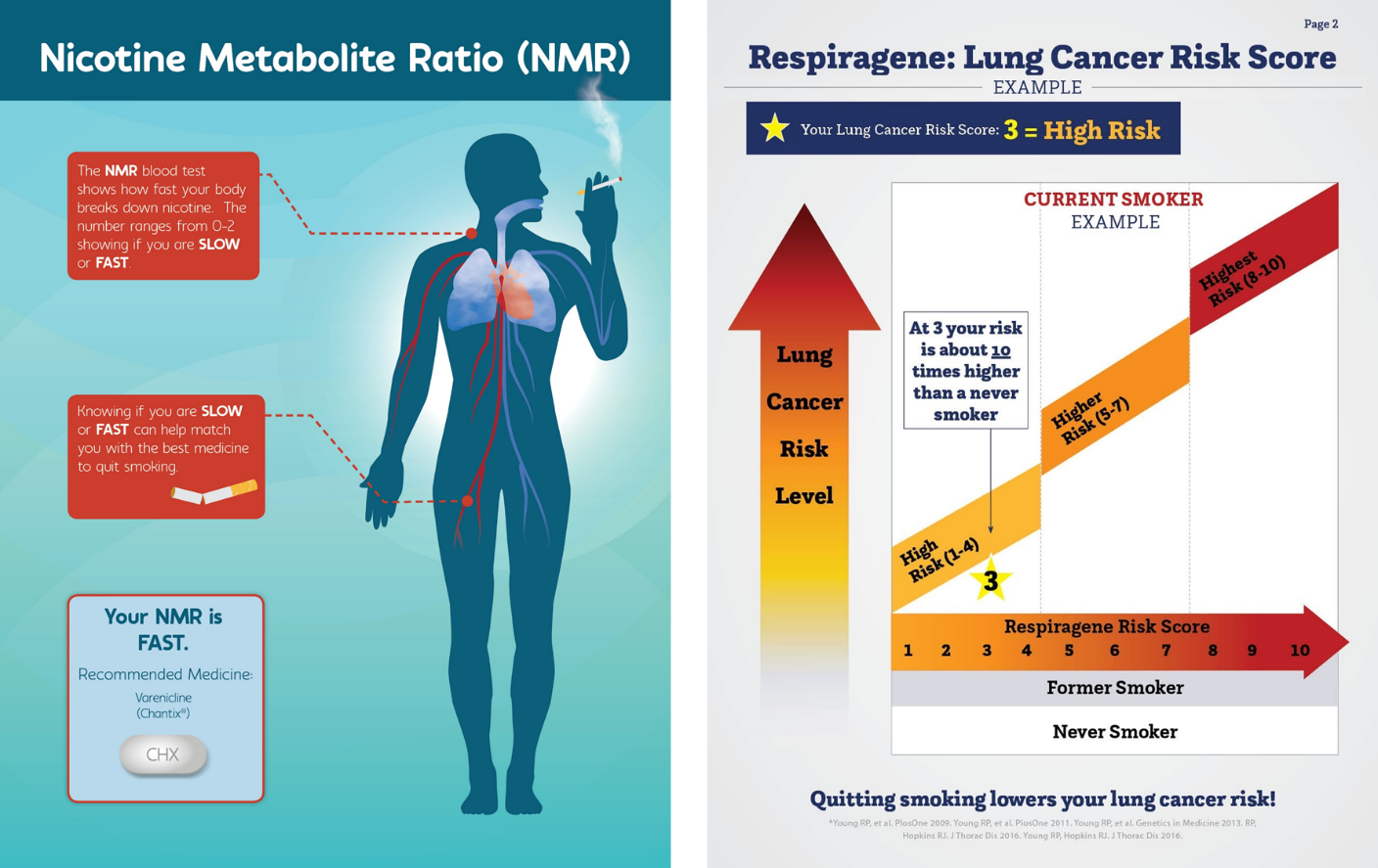


*Note:* Infographics were co-developed with a community advisory board that included people who smoke as well as do not smoke, as detailed in [35]. MIC: Metabolism-Informed Care; PRS: Polygenic Risk Score.
